# 5-Hydr­oxy-2-methyl-4*H*-pyran-4-one

**DOI:** 10.1107/S1600536809003158

**Published:** 2009-01-31

**Authors:** Muhammad Ashraf Shaheen, Christian G. Hartinger, M. Nawaz Tahir, Ahmad Awan Shafiq, Bernhard K. Keppler

**Affiliations:** a Department of Chemistry, University of Sargodha, Sargodha, Pakistan; b Institute of Inorganic Chemistry, University of Vienna, Whaeringer Strasse 42, A-1090 Vienna, Austria; c Department of Physics, University of Sargodha, Sargodha, Pakistan; d PAEC, PO Box No. 1114, Islamabad, Pakistan

## Abstract

The title compound, C_6_H_6_O_3_, is a member of the pyrone family. The mol­ecules are planar (r.m.s. deviation of the asymmetric unit is 0.0248 Å, whereas that of the dimer is 0.0360 Å) and they are dimerized due to inter­molecular O—H⋯O hydrogen bonds. The dimers are connected to each other through hydrogen bonds involving the CH_3_ group and the hydr­oxy O atom. There are π–π inter­actions between the centroids of the pyrone rings at a distance of 3.8552 (13) Å. A C—H⋯π inter­action also exists between the carbonyl group and the centroid *CgA* of the pyrone ring, with O⋯*CgA* = 3.65 (1) Å and C⋯*CgA* =  4.363 (2) Å.

## Related literature

For general background, see: Aytemir *et al.* (1999 ▶); Erol & Yulug (1999 ▶). For studies involving metal complexes of allomaltol, see: Ma *et al.* (2004 ▶); Shaheen *et al.* (2008 ▶, 2008*a* ▶). For crystal structures of related compounds, see: Tak *et al.* (1994 ▶); Rahman *et al.* (1997 ▶).
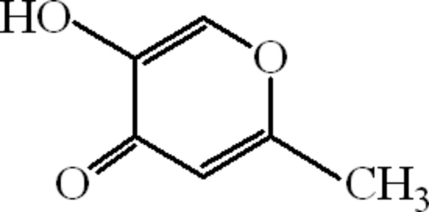


## Experimental

### 

#### Crystal data

C_6_H_6_O_3_
*M* *_r_* = 126.11
Triclinic, 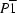

*a* = 5.4467 (4) Å
*b* = 7.3301 (5) Å
*c* = 7.6945 (5) Å
α = 105.354 (3)°
β = 98.416 (4)°
γ = 100.008 (4)°
*V* = 285.68 (4) Å^3^
*Z* = 2
Mo *K*α radiation
μ = 0.12 mm^−1^
*T* = 296 (2) K
0.22 × 0.20 × 0.10 mm

#### Data collection

Bruker Kappa APEXII CCD diffractometer
Absorption correction: multi-scan (*SADABS*; Bruker, 2005 ▶) *T*_min_ = 0.970, *T*_max_ = 0.986
6426 measured reflections
1504 independent reflections
713 reflections with *I* > 2σ(*I*)
*R*_int_ = 0.045

#### Refinement

*R*[*F* ^2^ > 2σ(*F* ^2^)] = 0.053
*wR*(*F* ^2^) = 0.131
*S* = 1.00
1504 reflections
87 parameters
H atoms treated by a mixture of independent and constrained refinement
Δρ_max_ = 0.18 e Å^−3^
Δρ_min_ = −0.20 e Å^−3^

### 

Data collection: *APEX2* (Bruker, 2007 ▶); cell refinement: *APEX2*; data reduction: *SAINT* (Bruker, 2007 ▶); program(s) used to solve structure: *SHELXS97* (Sheldrick, 2008 ▶); program(s) used to refine structure: *SHELXL97* (Sheldrick, 2008 ▶); molecular graphics: *ORTEP-3 for Windows* (Farrugia, 1997 ▶) and *PLATON* (Spek, 2003 ▶); software used to prepare material for publication: *WinGX* (Farrugia, 1999 ▶) and *PLATON*.

## Supplementary Material

Crystal structure: contains datablocks global, I. DOI: 10.1107/S1600536809003158/at2715sup1.cif

Structure factors: contains datablocks I. DOI: 10.1107/S1600536809003158/at2715Isup2.hkl

Additional supplementary materials:  crystallographic information; 3D view; checkCIF report

## Figures and Tables

**Table 1:** Hydrogen-bond geometry (Å, °)

*D*—H⋯*A*	*D*—H	H⋯*A*	*D*⋯*A*	*D*—H⋯*A*
O2—H2⋯O3	0.87 (3)	2.46 (2)	2.7853 (19)	103.1 (18)
O2—H2⋯O3^i^	0.87 (3)	1.83 (3)	2.635 (2)	152 (2)
C6—H6*A*⋯O2^ii^	0.96	2.42	3.378 (3)	173

Symmetry codes: (i) 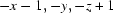; (ii) 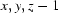; (iii) 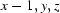.

**Fig. 1.**
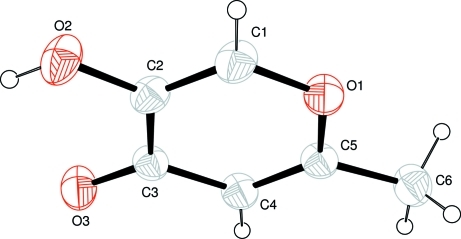
ORTEP drawing of the title compound, with the atom numbering scheme. The thermal ellipsoids are drawn at the 30% probability level. H atoms are shown by small circles of arbitrary radii.

**Fig. 2.**
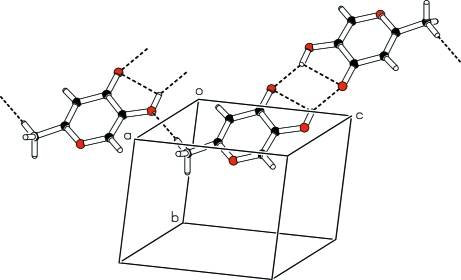
The packing figure (PLATON: Spek, 2003) which shows that the title compound is dimersed and dimers are connected through H-bonds in helical way.

## supplementary crystallographic information

### Comment

A variety of compounds with a 4(1*H*)-pyridinone structure have been synthesized and their biological activities studied extensively (Aytemir *et al.*, 1999; Erol & Yulug, 1999). Allomaltol, the title compound (I), (Fig. 1) and its derivatives have been exploited as iron chelators (Ma *et al.*, 2004). Ruthenium and osmium complexes of allomaltol were found to be effective in catalyzing the hydration of chloroacetonitriles (Shaheen *et al.*, 2008, 2008*a*).

The crystal structures of 3-Hydroxy-4-pyrone (Tak *et al.*, 1994) has been published which have same heterocyclic ring as of title compound. 3-Hydroxy-2-methyl-4*H*-pyran-4-one (Rahman *et al.*, 1997) has also been published which is chemical isomer of (I) but have different position of CH_3_. The title compound has been prepared for various purposes such as complexation and as an intermediate ligand.

The heterocyclic ring is not regular as it has two C—C [1.426 (3), 1.446 (3) Å], two C═C [1.323 (3), 1.334 (3) Å] and two C—O [1.352 (3), 1.358 (3) Å], bonds respectively. Due to intra as well as intermolecular H-bonds (Table 1), the molecules are dimerized with central four-membered [O···H···O···H] ring, (Fig. 2). The dimers are linked to each other through H-bond between CH_3_ and hydroxy groups. The molecules may be stabilized due to π–π interaction between the centroids of the ring A (O1/C1–C5). The distance between the centroids of CgA and CgA^i^ [Symmetry code: i = -*x*, 1 - *y*, 1 - *z*] is 3.8552 (13) Å. There exist a C3═O3···π interaction (Table 1), as well.

### Experimental

A mixture of 2-chloromethyl-5-hydroxy-4-pyron (1.0 g, 0.6 mmol) and zinc dust (0.8 g, 12 mmol) in water (20 ml) was stirred for 30 min at 323 K. Concentrated HCl (6 ml) was added dropwise to dissolve the zinc dust completely and the mixture was stirred for 3 h at 353 K. The reaction mixture was transferred to ice–water and extracted with dichloromethane, dried with anhydrous Na_2_SO_4_ and evaporated to dryness. The crystals of the title compound were obtained by recrystallizing the crude product in isopropanol.

### Refinement

The coordinates of H atom of hydroxy group were refined. H atoms were positioned geometrically, with C—H = 0.93 and 0.96 Å for aromatic and methyl H, and constrained to ride on their parent atoms, with *U*_iso_(H) = *xU*_eq_(C, O), where *x* = 1.5 for methyl H, and *x* = 1.2 for other H atoms.

### Crystal data

**Table:**

C_6_H_6_O_3_	*Z* = 2
*M**_r_* = 126.11	*F*(000) = 132
Triclinic, *P*1	*D*_x_ = 1.466 Mg m^−^^3^
Hall symbol: -P 1	Mo *K*α radiation, λ = 0.71073 Å
*a* = 5.4467 (4) Å	Cell parameters from 1504 reflections
*b* = 7.3301 (5) Å	θ = 2.8–29.1°
*c* = 7.6945 (5) Å	µ = 0.12 mm^−^^1^
α = 105.354 (3)°	*T* = 296 K
β = 98.416 (4)°	Prismatic, colourless
γ = 100.008 (4)°	0.22 × 0.20 × 0.10 mm
*V* = 285.68 (4) Å^3^	

### Data collection

**Table:**

Bruker Kappa APEXII CCD diffractometer	1504 independent reflections
Radiation source: fine-focus sealed tube	713 reflections with *I* > 2σ(*I*)
graphite	*R*_int_ = 0.045
Detector resolution: 7.40 pixels mm^-1^	θ_max_ = 29.1°, θ_min_ = 2.8°
ω scans	*h* = −7→7
Absorption correction: multi-scan (*SADABS*; Bruker, 2005)	*k* = −9→9
*T*_min_ = 0.970, *T*_max_ = 0.986	*l* = −10→10
6426 measured reflections	

### Refinement

**Table:**

Refinement on *F*^2^	Secondary atom site location: difference Fourier map
Least-squares matrix: full	Hydrogen site location: inferred from neighbouring sites
*R*[*F*^2^ > 2σ(*F*^2^)] = 0.053	H atoms treated by a mixture of independent and constrained refinement
*wR*(*F*^2^) = 0.131	*w* = 1/[σ^2^(*F*_o_^2^) + (0.0523*P*)^2^ + 0.0219*P*] where *P* = (*F*_o_^2^ + 2*F*_c_^2^)/3
*S* = 1.00	(Δ/σ)_max_ < 0.001
1504 reflections	Δρ_max_ = 0.18 e Å^−^^3^
87 parameters	Δρ_min_ = −0.20 e Å^−^^3^
0 restraints	Extinction correction: *SHELXL97* (Sheldrick, 2008), Fc^*^=kFc[1+0.001xFc^2^λ^3^/sin(2θ)]^-1/4^
Primary atom site location: structure-invariant direct methods	Extinction coefficient: 0.053 (14)

### Special details

**Table:**

Geometry. Bond distances, angles *etc*. have been calculated using the rounded fractional coordinates. All su's are estimated from the variances of the (full) variance-covariance matrix. The cell e.s.d.'s are taken into account in the estimation of distances, angles and torsion angles
Refinement. Refinement of *F*^2^ against ALL reflections. The weighted *R*-factor *wR* and goodness of fit *S* are based on *F*^2^, conventional *R*-factors *R* are based on *F*, with *F* set to zero for negative *F*^2^. The threshold expression of *F*^2^ > σ(*F*^2^) is used only for calculating *R*-factors(gt) *etc*. and is not relevant to the choice of reflections for refinement. *R*-factors based on *F*^2^ are statistically about twice as large as those based on *F*, and *R*- factors based on ALL data will be even larger.

### Fractional atomic coordinates and isotropic or equivalent isotropic displacement parameters (Å^2^)

**Table:**

	*x*	*y*	*z*	*U*_iso_*/*U*_eq_	
O1	0.2557 (3)	0.3634 (2)	0.37275 (16)	0.0542 (5)	
O2	−0.1534 (3)	0.1916 (2)	0.64758 (18)	0.0641 (6)	
O3	−0.4440 (3)	0.0215 (2)	0.29282 (18)	0.0631 (6)	
C1	0.1595 (4)	0.3242 (3)	0.5153 (3)	0.0554 (8)	
C2	−0.0705 (4)	0.2171 (3)	0.4961 (3)	0.0467 (7)	
C3	−0.2315 (4)	0.1292 (3)	0.3153 (3)	0.0455 (7)	
C4	−0.1223 (4)	0.1770 (3)	0.1711 (3)	0.0486 (7)	
C5	0.1096 (4)	0.2896 (3)	0.2016 (3)	0.0463 (7)	
C6	0.2424 (4)	0.3492 (4)	0.0642 (3)	0.0624 (8)	
H1	0.26084	0.37532	0.63280	0.0664*	
H2	−0.299 (5)	0.111 (4)	0.628 (3)	0.0769*	
H4	−0.21633	0.12780	0.05123	0.0584*	
H6A	0.13508	0.29403	−0.05590	0.0936*	
H6B	0.28060	0.48817	0.09427	0.0936*	
H6C	0.39779	0.30418	0.06523	0.0936*	

### Atomic displacement parameters (Å^2^)

**Table:**

	*U*^11^	*U*^22^	*U*^33^	*U*^12^	*U*^13^	*U*^23^
O1	0.0501 (9)	0.0655 (10)	0.0377 (8)	−0.0005 (7)	−0.0002 (6)	0.0131 (7)
O2	0.0703 (11)	0.0723 (12)	0.0356 (8)	−0.0115 (9)	0.0030 (7)	0.0143 (8)
O3	0.0506 (10)	0.0853 (12)	0.0440 (8)	−0.0041 (9)	0.0051 (7)	0.0182 (8)
C1	0.0612 (15)	0.0605 (15)	0.0346 (10)	0.0001 (12)	−0.0004 (10)	0.0117 (10)
C2	0.0540 (14)	0.0473 (13)	0.0343 (10)	0.0071 (11)	0.0035 (9)	0.0101 (9)
C3	0.0410 (13)	0.0519 (14)	0.0391 (11)	0.0078 (11)	0.0038 (9)	0.0099 (9)
C4	0.0457 (13)	0.0612 (15)	0.0327 (9)	0.0077 (11)	0.0027 (9)	0.0089 (10)
C5	0.0454 (13)	0.0544 (14)	0.0343 (10)	0.0090 (11)	0.0033 (9)	0.0091 (9)
C6	0.0547 (14)	0.0819 (17)	0.0488 (12)	0.0074 (12)	0.0110 (10)	0.0210 (12)

### Geometric parameters (Å, °)

**Table:**

O1—C1	1.358 (3)	C4—C5	1.334 (3)
O1—C5	1.352 (3)	C5—C6	1.480 (3)
O2—C2	1.356 (3)	C1—H1	0.9300
O3—C3	1.243 (3)	C4—H4	0.9300
O2—H2	0.87 (3)	C6—H6A	0.9600
C1—C2	1.323 (3)	C6—H6B	0.9600
C2—C3	1.446 (3)	C6—H6C	0.9600
C3—C4	1.426 (3)		
			
O1···O3^i^	3.200 (2)	C2···O1^iii^	3.350 (3)
O1···O1^ii^	3.078 (2)	C2···O2^vi^	3.405 (3)
O1···C2^iii^	3.350 (3)	C2···C1^iii^	3.501 (3)
O2···O3	2.7853 (19)	C2···C2^vi^	3.415 (3)
O2···C6^iv^	3.378 (3)	C6···O2^ix^	3.378 (3)
O2···O3^v^	2.635 (2)	C3···H2^v^	3.00 (3)
O2···C2^vi^	3.405 (3)	C4···H6C^vii^	3.0000
O3···O2^v^	2.635 (2)	H2···O3	2.46 (2)
O3···O1^vii^	3.200 (2)	H2···O3^v^	1.83 (3)
O3···O2	2.7853 (19)	H2···C3^v^	3.00 (3)
O2···H6B^iii^	2.9000	H4···H6A	2.4500
O2···H6A^iv^	2.4200	H4···O3^viii^	2.8200
O3···H2	2.46 (2)	H6A···O2^ix^	2.4200
O3···H2^v^	1.83 (3)	H6A···H4	2.4500
O3···H4^viii^	2.8200	H6B···O2^iii^	2.9000
C1···C1^iii^	3.387 (3)	H6C···C4^i^	3.0000
C1···C2^iii^	3.501 (3)		
			
C1—O1—C5	118.57 (18)	O1—C5—C4	121.3 (2)
C2—O2—H2	116.2 (15)	O1—C1—H1	118.00
O1—C1—C2	123.6 (2)	C2—C1—H1	118.00
O2—C2—C3	120.55 (19)	C3—C4—H4	119.00
C1—C2—C3	120.2 (2)	C5—C4—H4	119.00
O2—C2—C1	119.3 (2)	C5—C6—H6A	109.00
O3—C3—C4	124.7 (2)	C5—C6—H6B	109.00
C2—C3—C4	113.9 (2)	C5—C6—H6C	109.00
O3—C3—C2	121.4 (2)	H6A—C6—H6B	109.00
C3—C4—C5	122.5 (2)	H6A—C6—H6C	109.00
O1—C5—C6	111.28 (19)	H6B—C6—H6C	109.00
C4—C5—C6	127.5 (2)		
			
C5—O1—C1—C2	0.0 (3)	C1—C2—C3—O3	177.0 (2)
C1—O1—C5—C4	−1.4 (3)	C1—C2—C3—C4	−2.6 (3)
C1—O1—C5—C6	179.1 (2)	O3—C3—C4—C5	−178.3 (2)
O1—C1—C2—O2	−177.69 (19)	C2—C3—C4—C5	1.3 (3)
O1—C1—C2—C3	2.0 (4)	C3—C4—C5—O1	0.7 (4)
O2—C2—C3—O3	−3.3 (3)	C3—C4—C5—C6	−179.9 (2)
O2—C2—C3—C4	177.2 (2)		

Symmetry codes: (i) *x*+1, *y*, *z*; (ii) −*x*+1, −*y*+1, −*z*+1; (iii) −*x*, −*y*+1, −*z*+1; (iv) *x*, *y*, *z*+1; (v) −*x*−1, −*y*, −*z*+1; (vi) −*x*, −*y*, −*z*+1; (vii) *x*−1, *y*, *z*; (viii) −*x*−1, −*y*, −*z*; (ix) *x*, *y*, *z*−1.

### Hydrogen-bond geometry (Å, °)

**Table:**

*D*—H···*A*	*D*—H	H···*A*	*D*···*A*	*D*—H···*A*
O2—H2···O3	0.87 (3)	2.46 (2)	2.7853 (19)	103.1 (18)
O2—H2···O3^v^	0.87 (3)	1.83 (3)	2.635 (2)	152 (2)
C6—H6A···O2^ix^	0.9600	2.4200	3.378 (3)	173.00
C3—O3···CgA^vii^	1.243 (3)	3.6465 (19)	4.363 (2)	117.56 (13)

Symmetry codes: (v) −*x*−1, −*y*, −*z*+1; (ix) *x*, *y*, *z*−1; (vii) *x*−1, *y*, *z*.
